# Determination of Polyphenols in *Ilex kudingcha* and *Insect Tea* (Leaves Altered by Animals) by Ultra-high-performance Liquid Chromatography-Triple Quadrupole Mass Spectrometry (UHPLC-QqQ-MS) and Comparison of Their Anti-Aging Effects

**DOI:** 10.3389/fphar.2020.600219

**Published:** 2021-01-21

**Authors:** Jianfei Mu, Fuping Yang, Fang Tan, Xianrong Zhou, Yanni Pan, Xingyao Long, Xin Zhao

**Affiliations:** ^1^ Chongqing Collaborative Innovation Center for Functional Food, Chongqing University of Education, Chongqing, China; ^2^ College of Food Science, Southwest University, Chongqing, China; ^3^ Tuberculosis Section III, Chongqing Public Health Medical Treatment Center, Chongqing, China; ^4^ Department of Public Health, Our Lady of Fatima University, Valenzuela, Philippines; ^5^ Department of Food Science and Biotechnology, Cha University, Seongnam, South Korea

**Keywords:** ilex kudingcha, insect tea, polyphenols, aging, UHPLC-QqQ-MS

## Abstract

*Ilex kudingcha* C.J. Tseng tea and insect tea, as traditional Chinese teas, are favored for their original craftsmanship, unique flavor, and biological functionality. In this study, ultra high-performance liquid chromatography-triple quadrupole mass spectrometry (UHPLC-QqQ-MS) was used to analyze the bioactive components of the extracts of *Ilex kudingcha* and *insect tea*, and D-galactose-induced aging mice were used to compare the *in vivo* anti-aging effects of *Ilex kudingcha* and *insect tea* extracts. The results were remarkable, UHPLC-QqQ-MS analysis showed that ITP contains 29 ingredients, while IKDCP contains 26 ingredients. However, due to the large differences in the content of the main chemical components in IKDCP and ITP, the effects are equally different. At the same time, the *in vivo* research results suggesting that the anti-aging effects of IKDCP and ITP (500 mg/kg) include the regulation of viscera indices of major organs; improvement in liver, skin, and spleen tissue morphology; decreased production of inflammatory cytokines; up regulation of *SOD, CAT, GSH, GSH-PX*, and *T-AOC* and down regulation of *NO* and *MDA* levels in serum and liver tissue; reductions in the concentration of pro-inflammatory factors, and increases in the concentration of anti-inflammatory factor. *RT-qPCR* and western blot assay also showed that IKDCP and ITP affect anti-aging by regulating the gene and protein expression of *GSH-PX, GSH1, SOD1, SOD2*, and *CAT*. The overall results indicate that ITP is more effective in treating oxidative damage in aging mice induced by D-galactose. Thus, ITP appears to be an effective functional drink owing to its rich nutritional components and anti-aging activities.

## Introduction

Aging is a comprehensive and complex physiological and pathological process of the decline of body molecules, cells, organs, and other functions, and is the result of multiple factors (Finkel, 2000). At present, the mechanism of aging is unclear. The most widely accepted theory is that of free radicals, which was first proposed by Harman in 1956 (Harman, 1956), who noted that free radicals are always generated in the body. Under normal circumstances, the body is located in a dynamic equilibrium that continuously generates and scavenges free radicals. With the increase in age, the amount of oxygen free radicals in the body accumulates, and the body is not in equilibrium, which will destroy the cell structure, and cause lipid peroxidation and cross-linking of protein and nucleic acid molecules. In addition, problems such as decreased biological enzymatic activity as well as DNA gene mutations or abnormal replication occur (Clement et al., 2019; Sánchez-Rodríguez et al., 2019). Those processes interfere with the normal metabolic activities of the human body, cause diseases, and accelerate human aging.

Based on the physiological characteristics of various experimental animals, establishing animal models to emulate clinical aging symptoms has become an important method for studying aging mechanisms and evaluating anti-aging drugs (Das et al., 2001). At present, although the etiology and mechanism of aging have not been clarified, a variety of aging animal models have been introduced in the study of anti-aging pathogenesis. Most of these models are designed according to modern aging theory, and can be roughly divided into ozone damage aging models, SAM mouse rapid aging models, thymic aging models, natural animal aging models, and D-galactose aging models (Song et al., 1999; Wei et al., 2005). In D-galactose-induced aging animal models, the pathological biochemical characteristics of mouse tissues and organs are similar to those of natural aging animal models. This model gets the advantages of easy modeling and good reproducibility. The principle of action is tantamount to inject animals with D-galactose for a continuous period of time to increase the galactose concentration in the animal body. D-Galactose generates glyoxal and hydrogen peroxide under the action of galactose oxidase, which increases reactive oxygen species and causes lipid peroxidation and an increase in superoxide anion free radicals. This causes further lipid peroxidation and an increase in superoxide anion free radicals, as well as behavioral abnormalities, biochemical changes, immune dysfunction, histopathological changes, abnormal gene expression and regulation, and decreased cell growth and reproductive ability in D-galactose aging model mice, which eventually leads to aging of the organism (Lin L. et al., 2018; Bo-Htay et al., 2018). Vitamin C is a natural antioxidant and the most common functional antioxidant. Many researchers have conducted research on the antioxidant effect of VC. Its antioxidant effect is remains relatively stable and obvious. The use of VC as a positive control for aging models has been widely verified and used (Yi et al., 2019; Forman et al., 2020; Li C. et al., 2019b).

Non-Camellia Tea was proposed by Xiao Peigen in 2011 (Ren et al., 2019). There are mainly four types, Jinyinhua tea, Juhua tea, small leaf kuding tea, and Vine tea. They are long-term drinking form folks all over the world, and non-camellia varieties for the prevention of chronic metabolic diseases. In addition, ‘Sweet tea’, the leaves of lithocarpus polystachyus rehd, is also a kind of Non-Camellia Tea, a new natural source of biologically active dihydrochalcone with various health benefits (Shang et al., 2020). *Ilex kudingcha* C.J. Tseng, as a non-camellia tea, is a traditional Chinese tea that is widely used, second only to black/green tea (Camellia sinensis). It is a folk traditional medicinal plant in southwest of China, rich in various effective ingredients, and has a long history of folk use. In recent years, it has become increasingly popular with consumers, and is known as “beauty tea”, “yishou tea”, “green gold” and other names (Yi et al., 2019). The chemical composition of *Ilex kudingcha* processed by holly leaves is relatively complex, mainly including *triterpenes* and *glycosides*, *flavonoids*, *polyphenols*, *amino acids*, *volatile oils* and *polysaccharides* and other effective substances. It has health care and medicinal effects of preventing and treating cardiovascular diseases, anti-oxidation, lowering blood fat, anti-virus and lowering blood sugar (Gan et al., 2018; Xie et al., 2018; Zhou et al., 2018).

*Insect tea* is a unique drink made from *Ilex kudingcha* that is digested by enzymes and microorganisms in larvae produced by insects attracted by the fermented of boiled rice water (Zhu et al. 2019). In the 16th century, *insect tea*, as a traditional drink of ethnic minorities in southwest China and one of China’s traditional export commodities, was included in Li Shizhen’s Compendium of Materia Medica -Materia Medica classics (Xu et al., 2013). Studies have shown that insect tea contains crude protein, amino acids, polyphenols, tannins, vitamin C, and the mineral elements *K*, *Mg*, *Cu*, *Fe*, *P*, and *Zn* and other chemical components, with a variety of beneficial health and pharmacological effects, such as anti-oxidation, anti-tumor, and lowering of blood pressure and blood lipids (Mao et al., 2019; Mao et al., 2020).

In recent years, there has been continuous research on *Ilex kudingcha*, but there is less research on insect tea made from *Ilex kudingcha*. Some studies have shown that the polyphenols in *Ilex kudingcha* play an important role in its health functions, such as anti-oxidant, anti-inflammatory, and anti-cancer (Song et al., 2016; Zhai et al., 2017). Studies have also shown that *insect tea* contains a variety of functional ingredients, including *polyphenols*, *minerals*, and *amino acids*, with approximately 10% polyphenols (Liao et al., 2016; Zhao et al., 2018). In this context, the aging model of mice induced by D-galactose was used to evaluate the protective effect of *Ilex kudingcha* polyphenol extract (IKDCP) and *insect tea* polyphenol extract (ITP) on aging, and determine their bioactive compounds.

## Materials and Methods

### Extraction of Ilex Kudingcha Polyphenol and Insect Tea Polyphenol

For extraction, 200 g of crushed *Ilex kudingcha* leaves (Hainan Coconut Island Agricultural E-commerce Co., Ltd., Hainan, China) and *insect tea* (Guizhou Zouzizhi Tea Development Co., Ltd, Guizhou, China) samples were weighed. Next, 1800 ml of distilled water was added at a ratio of 1:9 (v/v), and extraction was conducted at 90°C for 30 min. After a repeat of the extraction, the two extracts were combined, the filtrate was enclosed in a round-bottomed flask, and the liquid was dried by rotary evaporation to obtain IKDCP and ITP (Zhang J. et al., 2020; Zhu et al., 2019).

### Determination of Ilex Kudingcha Polyphenol and Insect Tea Polyphenol Composition

HPLC-grade methyl alcohol was purchased from Adamas Reagents, Ltd. (Shanghai, China). All the standards used for identification by UPLC-QqQ-MS were purchased from Shanghai Yuanye Biotechnology Co., Ltd. (Shanghai, China). Ultrapure water was available from Hangzhou Wahaha Group Co., Ltd. (Hangzhou, China).

The chemical composition of IKDCP and ITP was analyzed by ultra-high-pressure liquid chromatography (UPLC, Agilent, CA, USA), and triple quadruple mass spectrometry (G6460C-MS, Agilent, CA, USA) was conducted using a spectrophotometer equipped with an electrospray ionization (ESI) source. The ZORBAX Eclipse plus C18 column (100 × 2.1 mm id., 1.8 μm, Agilent, Waldbronn, Germany) was used to separate the compounds. The mobile phase consisted of a gradient of (A) 0.1% aqueous formic acid and (B) acetonitrile at a flow rate of 0.3 ml/min. The gradient elution was set as follows: 0–2 min, 20% B, 2–8 min, 20%–95% B, 8–13 min, 95% B, 13–15 min 20% B. Solutions of IKDCP and ITP were prepared by completely dissolving 100 mg of sample in 5 ml methanol. The sample injection volume was 5 μL, and the column temperature was 30°C. The ESI source was simultaneously operated in positive and negative ion mode, and the dynamic multiple reaction monitoring (MRM) data were acquired. The drying gas flow and temperature were 10 ml/min and 350°C, respectively (Qian et al., 2018; Rocchetti et al., 2018). All the compounds were identified by comparing the dynamic MRM information including retention time (RT), fragmentor voltages, collision energies (CE), and transitions with reference standards (Table 1). All analyses were performed three times in parallel, and the results are given in nanogram per Gram of dry weight.

**TABLE 1:** Identification of phenolic compounds using UHPLC-QqQ-MS/MS.

No	Compounds	RT	MS [M-H]^-^	MS/MS (m/z)	CE (V)	Fragmentor (V)
1	Kaempferol	0.325	286.9	211.0	40	140
2	Gallocatechin	2.979	307.3	138.8, 162.9	20	140
3	(−)-Gallocatechin	2.989	308.0	139.0, 136.7	15	150
4	Chlorogenic acid	4.376	355.3	162.8, 355.3	10	100
5	Cryptochlorogenic acid	4.377	355.0	162.8, 355.0	20	100
6	p-Coumaric acid	4.381	164.0	164.0	20	80
7	Gallocatechin gallate	4.931	459.0	139.0, 151.0	10	140
8	Epicatechin	5.132	291.0	138.8, 123.0	25	140
9	Epigallocatechin gallate	5.653	459.0	139.0, 111.0	10	140
10	Epicatechin gallate	5.905	443.4	122.9, 272.9	15	140
11	Rutin	6.545	609.1	300.1, 150.8	40	160
12	Isoquercitrin	6.581	465.4	302.9, 84.9	30	160
13	Vitexin	6.288	433.4	432.9, 312.9	30	120
14	Isovitexin	6.277	433.4	433.0	25	120
15	Hesperidin	6.535	611.6	303.0, 449.0	35	120
16	Gallic acid	6.774	170.0	170.0	15	100
17	Rosmarinic acid	6.584	361.3	163.0, 144.8	20	110
18	Narirutin	6.580	579.0	273.2, 435.0	20	160
19	Astragalin	6.823	449.0	286.9, 449.0	20	110
20	Protocatechuic acid	6.971	155.0	126.9, 155.0	15	100
21	Taxifolin	7.197	304.0	304.0	13	120
22	Quercetin	7.197	303.2	136.7, 175.8	25	130
23	Phloretin	7.242	275.3	106.9, 168.9	20	120
24	Hesperetin	7.307	303.0	176.9,152.8	30	130
25	Luteolin	7.34	287.1	152.8, 134.9	30	140
26	Caffeic acid	7.615	180.2	155.1	15	100
27	Baicalein	7.669	271.2	271.1 240.0	40	140
28	Cinnamic acid	8.719	149.2	149.2	15	80
29	Isochlorogenic acid A	10.034	517.2	517.2, 499.1	15	120
30	Isochlorogenic acid B	10.033	517.3	517.3, 162.6	25	100
31	Isochlorogenic acid C	10.033	517.5	517.0, 490.9	20	130
32	Phloridzin	11.826	473.4	87.8	20	150
33	Myricitrin	12.354	464.4	155.1	15	140

V: volt; RT: retention time (min); CE: collision energy.

### Establishment of the Aging Mouse Model

Fifty 6-week-old SPF grade female Kunming mice (Chongqing Medical University, Chongqing, China. License number: SCXK (yu) 2017–0001) were fed for 1 week to adapt to the environment, and then were divided into five groups: normal group, model group, vitamin C group (VC), IKDCP group, and ITP group, with 10 mice in each group. The mice in each group were fed normal maintenance food and drinking water. Additionally, the mice in the vitamin C group, IKDCP group, and ITP group received vitamin C, IKDCP, and ITP by intragastric administration at the concentrations of 100, 500, and 500 mg/kg once a day, respectively. The normal group and the model group were given corresponding doses of distilled water. The modeling for each group began on the 15th day of administration. Except for the normal group (who received an injection of an equal volume of normal saline), the other groups were treated with D-galactose (Sinopharm Chemical Reagent Co., Ltd., China) at 120 mg/kg intraperitoneally daily for 6 weeks to induce the aging model. After 8 weeks, all mice were fasted for 18 h, euthanized and then were removed from the eyeballs for blood. The heart, liver, skin, spleen, and kidney were gathered for subsequent experiments. The heart, liver, spleen, and kidney tissue was weighed, and the organ index was calculated by the formula: organ index (%) = organ mass (g)/mouse body mass (g) × 100 (Wu et al., 2020; Li et al., 2019).

### Determination of Serum Biochemical Indicators of Nitric Oxide, Total Antioxidant Capacity, Superoxide Dismutase, Catalase, Glutathione Peroxidase, Glutathione, and Mobile Device Assistant

Mouse blood was centrifuged at 4,000 rpm and 4°C for 10 min, and the supernatant was harvested. The serum content of *NO, T-AOC, SOD, CAT, GSH-PX, GSH*, and *MDA* of mice was determined by kit instructions (Nanjing Jiancheng Bioengineering Institute, Nanjing, Jiangsu, China).

### Measurement of IL-6, IL-12, TNF-α, and IL-1β in the Serum

Depending on the kit instructions, the serum levels of cytokines *IL-6, IL-12, TNF-α*, and *IL-1β* were determined by enzyme-linked immunosorbent assay (ELISA, Abcam, Cambridge, MA, USA).

### Determination of Nitric Oxide, Total Antioxidant Capacity, Superoxide Dismutase, Catalase, Glutathione Peroxidase, Glutathione and Mobile Device Assistant in the Liver Tissue

According to the kit instructions, 100 mg of liver tissue was removed, and 900 μL of physiological saline was added. The tissue was subsequently homogenized with a tissue homogenizer. The obtained tissue homogenate was centrifuged at 4,000 rpm and 4°C for 10 min, and the supernatant was harvested. The mouse liver tissue levels of *NO, T-AOC, SOD, CAT, GSH-PX, GSH*, and *MDA* were determined by kit instructions (Nanjing Jiancheng Bioengineering Institute, Nanjing, Jiangsu, China).

### Pathological Observation of Liver, Skin, and Spleen Tissues

Samples of liver, skin, and spleen tissues with the size of 0.5 cm^2^ were collected and fixed in tissue fixative for 48 h. The liver, skin, and spleen tissues were dehydrated, made transparent, waxed, embedded, sectioned, baked, sliced, and then stained with H&E. The tissue morphological changes were observed under an optical microscope and analyzed by image acquisition (BX43; Olympus, Tokyo, Japan).

### Quantitative PCR Assay

To extract the total RNA, 100 mg liver tissue, skin tissue, and spleen tissue of the mice were weighed, and 1 ml of TRIzol reagent (Invitrogen, Carlsbad, CA, USA) was separately added to the sample tissues, which were then pulverized using a tissue homogenizer. The concentration of the extracted total RNA was diluted to 1 μg/μL, and 1 μL of the diluted total RNA solution was submitted to reverse transcription using a kit to obtain the cDNA template. The reaction system was as follows: 1 μL of cDNA template was mixed with 10 μL of SYBR Green PCR Master Mix, 1 μL upstream and downstream primers, and 7 μL of enzyme-free water. The system was amplified under the following conditions: denatured at 95°C for 60 s, annealed at 60°C for 30 s, and extended at 72°C for 35 s, and cycled 40 times. Finally, the DNA was detected at 95°C for 30 s and 55°C for 35 s. The expression levels of the anti-oxidant genes *SOD1, SOD2, CAT, GSH-PX*, and *GSH1* were determined by RT-qPCR. The sequences of the related primers are shown in Table 2. GAPDH was used as an internal reference (StepOnePlus Real-Time PCR System; Thermo Fisher Scientific, Waltham, MA, USA), and the levels of the relevant genes were calculated according to the 2^−∆∆CT^ method (Li et al., 2019; Liao et al., 2016).

**TABLE 2:** Primer sequences.

Primer name	Forward primer	Reverse primer
GAPDH	5′-AGG​TCG​GTG​TGA​ACG​GAT​TTG-3′	5′-GGG​GTC​GTT​GAT​GGC​AAC​A-3′
SOD1	5′-AAC​CAG​TTG​TGT​TGT​CAG​GAC-3′	5′-CCA​CCA​TGT​TTC​TTA​GAG​TGA​GG-3′
SOD2	5′-CAG​ACC​TGC​CTT​ACG​ACT​ATG​G-3′	5′-CTC​GGT​GGC​GTT​GAG​ATT​GTT-3′
Catalase	5′-GGA​GGC​GGG​AAC​CCA​ATA​G-3′	5′-GTG​TGC​CAT​CTC​GTC​AGT​GAA-3′
GSH1	5′-GGG​TGA​AGC​ACA​AGA​AAG​AAG​G-3′	5′-TTG​GCT​GAG​GAG​CGA​AGA-3′
GSH-PX	5′-CCA​CCG​TGT​ATG​CCT​TCT​CC-3′	5′-AGA​GAG​ACG​CGA​CAT​TCT​CAA​T-3′

### Western Blot Analysis

Samples of 100 mg liver tissue, skin tissue, and spleen tissue of the mice were homogenized with 1 ml of RIPA buffer (Thermo Fisher Scientific) and 10 μL of PMSF, and then centrifuged at 12,000 rpm and 4°C for 10 min. The intermediate protein layer solution was collected, and a BCA kit was used for protein quantification. It was determined that the protein loading amount was 50 μg, and the loading volume was 10 μL. The concentration of each sample was diluted to 6 μg/μL, and the diluted protein was mixed with sample buffer at the ratio of 4:1 and heated at 100°C for 10 min. Then, acrylamide, resolving buffer, stacking buffer, distilled water, 10% APS, and TEMED (Thermo Fisher Scientific) were mixed in proportion to make a SDS-PAGE separation gel and stacking gel, and the mixed solution was poured between the gel plates. The samples and the prestained protein ladder (Thermo Fisher Scientific) were separately loaded into the sample wells of the gel plate, and the protein-loaded SDS-PAGE gel was subjected to vertical gel electrophoresis until the prestained protein ladder was separated. A PVDF membrane (Thermo Fisher Scientific) was activated with methanol for 1 min and then transmembrane operation. After that, the PVDF membrane was blocked with TBS containing 0.1% Tween-20 (TBST) solution containing 5% skim milk for 1 h, transferred to 5% skim milk containing primary antibody, and incubated at 25°C for 2 h. The membrane and the secondary antibody were incubated at 25°C for 1 h. The PVDF membrane was washed 5 times with TBST, treated with Supersignal West Pico PLUS, and then placed in the iBright FL1000 (Thermo Fisher Scientific) imaging system for observation (Liao et al., 2016; Qian et al., 2018).

### Statistical Analysis

The serum and tissue assays for each mouse were performed in three parallel experiments, and the average value was calculated. The data are presented as the mean ± standard deviation (SD). SPSS statistical software (SPSS Inc, Chicago, Illinois, USA) was used for data analysis, one-way analysis of variance (ANOVA) for Post-Hoc two-two comparison was used to determine whether there were significant differences among groups of data at the level of *p* < 0.05.

## Results

### Composition Analysis of Ilex Kudingcha Polyphenol and Insect Tea Polyphenol

**FIGURE 1:**
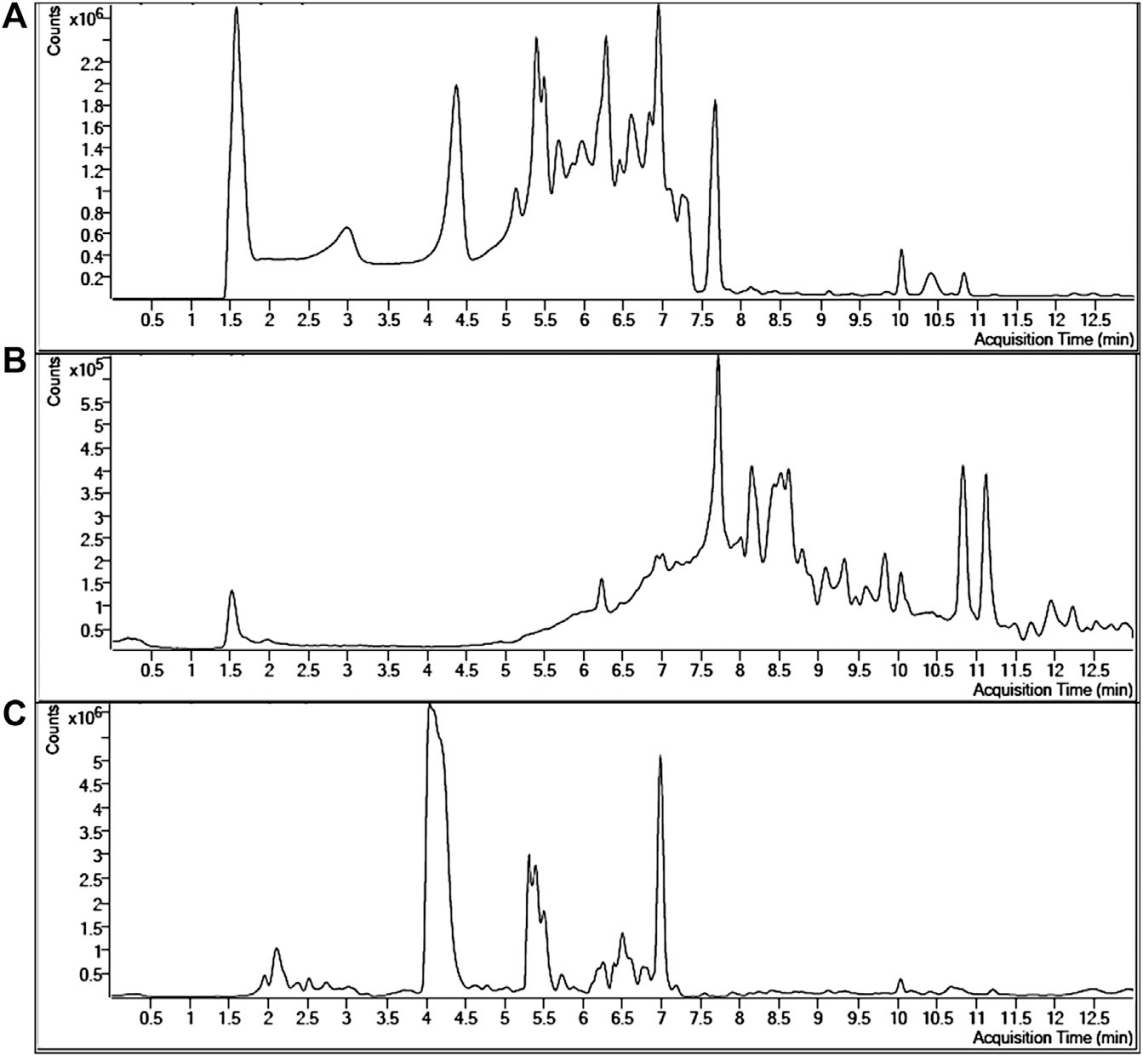
Extracted dynamic MRM chromatograms with retention times of standards of phenolic acids and flavonoids **(A)**, polyphenols in Insect tea **(B)** and polyphenols in *Ilex kuding tea* **(C)**.

In order to better understand the anti-oxidant effects of *Ilex kudingcha* and *insect tea*, the content of each polyphenol in IKDCP and ITP fragments was studied. We compared dynamic MRM information for 33 polyphenols (Figure 1). We found 29 and 26 polyphenol compounds in ITP and IKDCP. *Cryptochlorogenic acid* and *rutin* were abundant in IKDCP but could not be found in ITP. However, *epicatechin, epigallocatechin gallate, epicatechin gallate, isovitexin*, and *hesperetin* were detected in ITP, but not in IKDCP. Other polyphenols were detected in IKDCP and ITP, and the content of each substance was different (Table 3). In particular, *kaempferol, narirutin, protocatechuic acid, caffeic acid, phloretin, taxifolin, isochlorogenic acid A, isochlorogenic acid B*, and *phloridzin* were significantly higher in ITP than in IKDCP. In addition, *chlorogenic acid, cryptochlorogenic acid, p-coumaric acid, isoquercitrin*, and *hesperidin* accounted for 61.5% of the total content in IKDCP.

**TABLE 3:** Contents of chemical compounds in IKDCP and ITP.

No	Compounds	ITP (%)	IKDCP (%)
1	Kaempferol	8.54	1.17
2	Gallocatechin	0.00	0.00
3	(−)-Gallocatechin	0.00	0.00
4	Chlorogenic acid	0.009	29.1
5	Cryptochlorogenic acid	0.00	19.4
6	p-Coumaric acid	0.146	7.01
7	Gallocatechin gallate	0.034	0.006
8	Epicatechin	0.004	0.00
9	Epigallocatechin gallate	0.012	0.00
10	Epicatechin gallate	0.016	0.00
11	Rutin	0.00	4.04
12	Isoquercitrin	0.033	8.28
13	Vitexin	0.425	0.235
14	Isovitexin	0.650	0.00
15	Hesperidin	0.012	4.89
16	Gallic acid	1.10	1.29
17	Rosmarinic acid	0.031	0.005
18	Narirutin	26.7	7.28
19	Astragalin	0.135	1.83
20	Protocatechuic acid	2.89	0.337
21	Taxifolin	1.08	0.184
22	Quercetin	0.044	0.201
23	Phloretin	0.336	0.010
24	Hesperetin	0.005	0.00
25	Luteolin	0.062	0.495
26	Caffeic acid	5.55	0.165
27	Baicalein	0.162	0.010
28	Cinnamic acid	6.22	2.88
29	Isochlorogenic acid A	23.5	3.78
30	Isochlorogenic acid B	14.1	3.41
31	Isochlorogenic acid C	6.46	3.81
32	Phloridzin	1.40	0.119
33	Myricitrin	0.439	0.111

ND: not detected. Results are presented as the means ± SD of three replicates.

### Organ Indices

The mice in the normal group exhibited the highest cardiac index, liver index, spleen index, and kidney index, while the lowest indices were observed for those in the model group (Table 4). After the administration of IKDCP, ITP, and vitamin C, the organ indices of all aging mice increased. There was no significant difference in the cardiac index between the IKDCP group and the ITP group, but there were significant differences in the other organ indexes (*p* < 0.05). The indices of the ITP group were significantly higher than those of the IKDCP group, and were comparable to the indices of the normal group. It can be concluded that IKDCP and ITP can alleviate the reduction of heart, liver, spleen, and kidney indexes to a certain extent, delay the degradation of mouse organs, and exert an antagonistic effect on organ atrophy.

**TABLE 4:** Effect of IKDCP and ITP on the organ indices in mice.

Group	Cardiac index	Liver index	Spleen index	Kidney index
Normal	4.93 ± 0.60^a^	36.4 ± 2.4^a^	4.04 ± 0.66^b^	12.5 ± 0.9^a^
Model	4.50 ± 0.65^b^	32.5 ± 2.5^e^	2.56 ± 0.54^e^	9.19 ± 0.87^e^
VC	4.71 ± 0.49^a^	33.9 ± 2.4^b^	3.21 ± 0.48^d^	9.59 ± 0.82^b^
IKDCP	4.80 ± 0.47^a^	34.4 ± 2.9^d^	3.78 ± 0.72^c^	9.79 ± 0.91^d^
ITP	4.87 ± 0.52^a^	35.7 ± 2.5^c^	4.14 ± 0.61^a^	10.3 ± 0.8^c^

Values presented are the mean ± standard deviation (N = 10/group).

^a–e^Mean values with different letters over the same column are significantly different (*p* < 0.05) according to Duncan's multiple range test. VC: mice treated with vitamin C (100 mg/kg); IKDCP: mice treated with polyphenol of *Ilex kudingcha* (500 mg/kg); ITP: mice treated with polyphenol of insect tea (500 mg/kg).

### Serum Content of the Biochemical Indicators Nitric Oxide, Total Antioxidant Capacity, Superoxide Dismutase, Catalase, Glutathione Peroxidase, Glutathione, and Mobile Device Assistant in Mice

**FIGURE 2:**
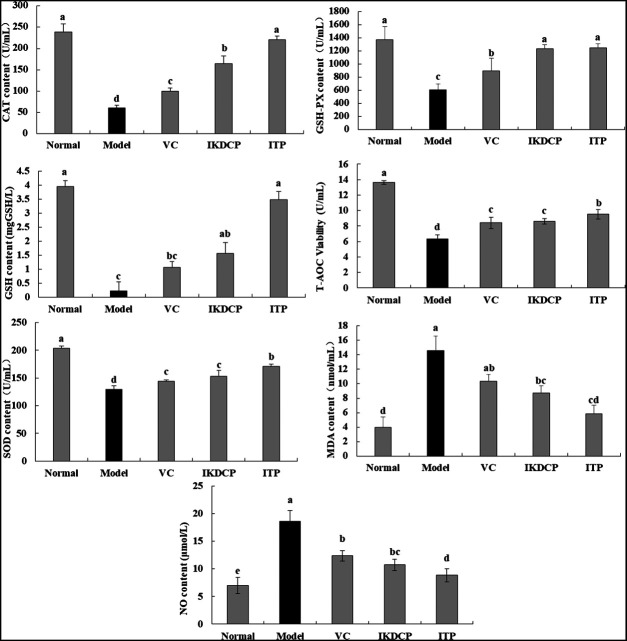
The content of NO, T-AOC, SOD, CAT, GSH-PX, GSH and MDA in serum of mice (N = 10). ^a–e^Mean values with different letters in the bar are significantly different (*p* < 0.05) according to Duncan’s multiple-range test. VC: mice treated with vitamin C (100 mg/kg); IKDCP: mice treated with polyphenol of *Ilex kudingcha* (500 mg/kg); ITP: mice treated with polyphenol insect tea (500 mg/kg).

Figure 2 indicates that the content of *T-AOC, SOD, CAT, GSH-PX,* and *GSH* in the serum of healthy mice (normal group) was the highest, while NO and MDA levels were lowest. The mice in the Aging (model group) exhibited a contrary trend. Compared with the model group, the content of *T-AOC, SOD, CAT, GSH-PX*, and *GSH* in the VC, ITP, and IKDCP groups was significantly increased (*p* < 0.05). In addition, the activity of *T-AOC, SOD, CAT, GSH-PX*, and *GSH* in the VC group was comparable with that in the IKDCP group, and the ITP group exhibited a higher content than the VC group with significant differences (*p* < 0.05). However, the content of *NO* and *MDA* was the lowest in the ITP group compared with the VC and IKDCP groups. Thus, the effect of ITP was stronger than that of VC and IKDCP.

### IL-6, IL-10, TNF-α, and IL-1β Concentrations in Serum

The serum cytokine detection assay showed that the serum levels of *IL-1β, IL-6*, and *TNF-α* in normal mice were lowest and that of *IL-10* was the highest (Table 5), while these indices in the model group exhibited a contrary tendency. Vitamin C, IKDCP, and ITP alleviated the effects of aging in mice. The serum levels of cytokines *IL-1β, IL-6*, and *TNF-α* in aging mice (model group) were significantly higher than those in mice of the normal group, and *IL-10* in the model group was significantly lower than that in normal mice without non-intervention (*p* < 0.05). The greatest decrease in *IL-1β, IL-6*, and *TNF-α* cytokines was attributed to the action of ITP, and its effect was significantly stronger than vitamin C and IKDCP.

**TABLE 5:** The levels of IL-1β, IL-6, IL-10 and TNF-α in serum of mice.

Group	TNF-α (pg/mL)	IL-6 (pg/mL)	IL-10 (pg/mL)	IL-1β (pg/mL)
Normal	895±54^d^	75.3±31.0^e^	247±32^a^	1.34E3±32.54^d^
Model	3.54E3±73.34^a^	163±15^a^	96.4±23.5^d^	1.96E3±24.76^a^
VC	2.17E3±63.53^b^	127±36^b^	164±19^c^	1.69E3±23.64^b^
IKDCP	2.33E3±83.52^b^	107±25^c^	174±36^c^	1.60E3±34.12^bc^
ITP	1.75E3±67.43^c^	91.5±12.0^d^	204±26^b^	1.55E3±53.37^c^

Values presented are the mean ± standard deviation (N = 10/group).

^a–e^ Mean values with different letters over the same column are significantly different (*p* < 0.05) according to Duncan's multiple range test. VC: mice treated with vitamin C (100 mg/kg); IKDCP: mice treated with polyphenol of *Ilex kudingcha* (500 mg/kg); ITP: mice treated with polyphenol of insect tea (500 mg/kg).

### Liver Tissue Levels of Superoxide Dismutase, Catalase, Glutathione Peroxidase, Glutathione, and Mobile Device Assistant

As shown in Table 6, the content of *SOD, CAT, GSH-PX*, and *GSH* was highest and *MDA* was lowest in the liver tissues of normal mice, while the aging mice (model group) exhibited the opposite trend. After treatment with vitamin C, IKDCP, and ITP, compared with the model group, the *SOD, CAT, GSH-PX*, and *GSH* content in each group significantly increased (*p* < 0.05), and the *MDA* content significantly decreased (*p* < 0.05). The strongest effect was ascribed to ITP, with indicators that were similar to those of the normal group.

**TABLE 6:** The content of CAT, GSH, GSH-PX, SOD and MDA in liver tissue of mice.

Group	CAT (U/mL)	GSH (mgGSH/gprot)	MDA (nmol/mgprot)	SOD (U/mL)	GSH-PX (U/mL)
Normal	16.4 ± 0.4^a^	3.25 ± 0.16^a^	3.51 ± 0.29^d^	36.9 ± 1.6^a^	1.13E3±59.65^a^
Model	11.8 ± 0.2^d^	1.58 ± 0.05^c^	7.77 ± 0.75^a^	22.9 ± 1.4^d^	579 ± 62^d^
VC	12.8 ± 0.4^c^	2.49 ± 0.08^b^	5.59 ± 0.36^b^	29.0 ± 1.5^c^	891 ± 54^c^
IKDCP	13.3 ± 0.5^b^	2.49 ± 0.17^b^	5.71 ± 0.74^b^	32.1 ± 1.0^b^	892 ± 85^c^
ITP	14.7 ± 0.3^b^	3.19 ± 0.24^a^	4.37 ± 0.43^c^	34.2 ± 2.9^ab^	1.07E3±25.83^ab^

Values presented are the mean ± standard deviation (N = 10/group).

^a–e^ Mean values with different letters over the same column are significantly different (*p* < 0.05) according to Duncan’s multiple range test. VC: mice treated with vitamin C (100 mg/kg); IKDCP: mice treated with polyphenol of *Ilex kudingcha* (500 mg/kg); ITP: mice treated with polyphenol of insect tea (500 mg/kg).

### Pathological Observation of Liver, Skin, and Spleen Tissue of Mice

**FIGURE 3:**
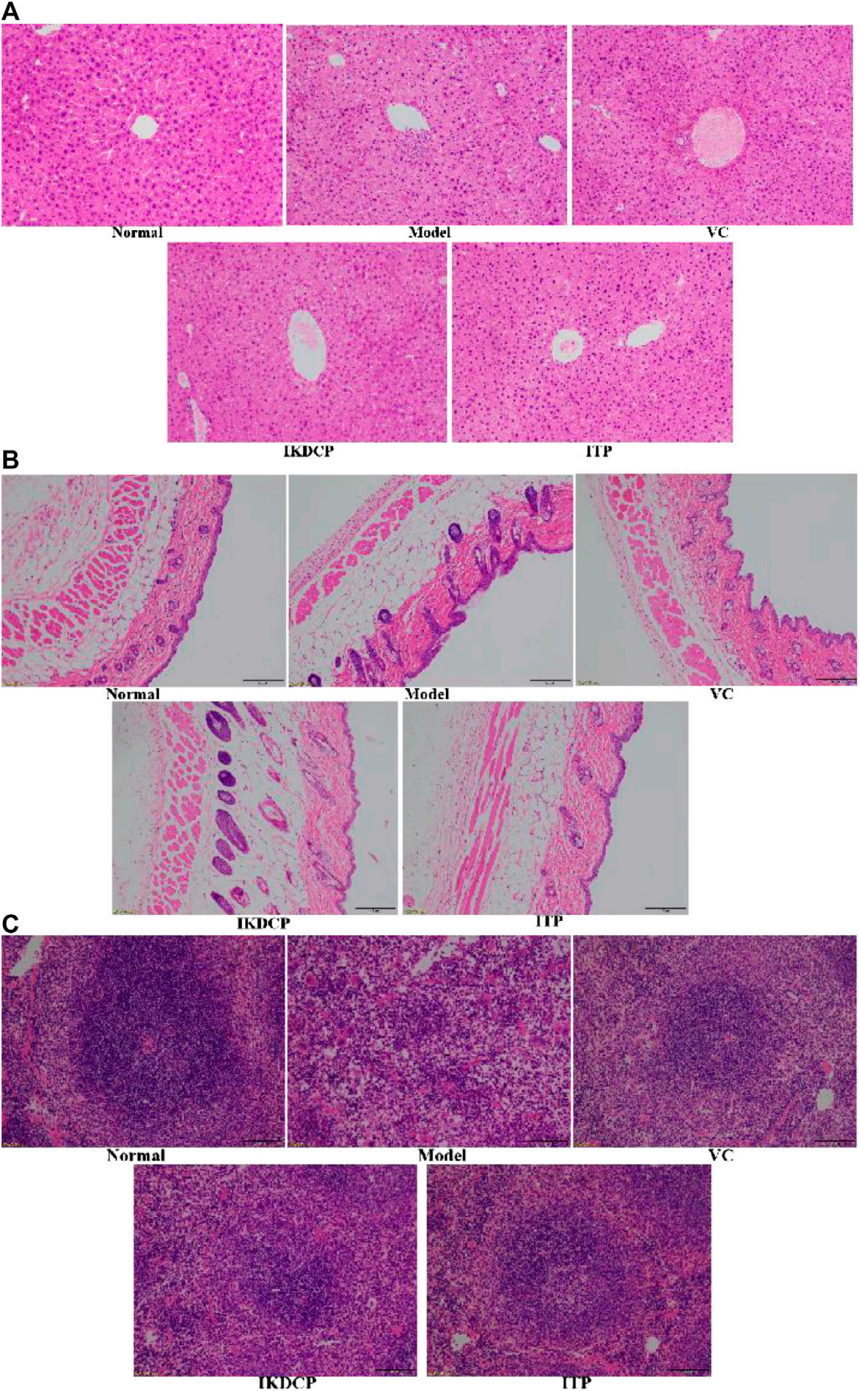
H&E pathological observation of liver tissue **(A)**, skin tissue **(B)** and spleen tissue **(C)** in mice. Magnification ×100. VC: mice treated with vitamin C (100 mg/kg); IKDCP: mice treated with polyphenol of *Ilex kudingcha* (500 mg/kg); ITP: mice treated with polyphenol of insect tea (500 mg/kg).

Normal mouse liver cells exhibited regular morphology, uniform size and staining, ordered hepatocyte cords, clear boundaries, and radial distribution centered on the central vein (Figure 3A). In the aging model group, the liver cells were unevenly arranged, the shape of the central vein was irregular, the arrangement of hepatocytes was disordered, the cell structure was destroyed, the boundaries were blurred, and the phenomenon of chromatin condensation was evident. However, the arrangement of mouse liver cell cords in mice treated with vitamin C, IKDCP, and ITP was basically orderly, and the morphology was slightly changed, which can alleviate cell necrosis and liver tissue incompleteness caused by D-galactose treatment in mice. The morphology of the liver tissue in the ITP treatment group resembled that of the normal group.

A pathological section of skin tissue as viewed under an optical microscope with the aging model mice induced by D-galactose is shown in Figure 3B. The normal mouse epidermal structure was clear and complete, with abundant collagen fibers in the dermal layer, clear boundaries between the dermal layer and the epidermal layer, and a normal number of fat vacuoles. Compared to normal mice, the aging mice exhibited less collagen fibers, increased fat vacuoles, dermal layer invasion, and blurred boundaries between dermis and epidermis. Report to the model group, there were fewer fat vacuoles in the vitamin C, IKDCP, and ITP groups, and the osmotic phenomenon was effectively alleviated. The epidermal and dermal boundaries were more discernible. The results showed that IKDCP and ITP can alleviate the skin tissue lesions of aging model mice induced by D-galactose, with a stronger effect by ITP resulting in levels that were closer to normal as compared to IKDCP.

The spleen structure of normal mice was clear and intact, the junction of the cortex and pulp was clear, and the cells were regularly arranged (Figure 3C). The damaged spleen structure disappeared, the shape was irregular, the red pulp myeloid sinus expanded and was filled with a large number of red blood cells, white pulp lymphocytes decreased, red pulp myelin became narrow, and the cells were sparsely arranged. After treatment with vitamin C, IKDCP, and ITP, the spleen morphology and structure were significantly improved, the boundaries of red pulp and white pulp were clearer, germinal centers were more obvious, and cells were uniformly arranged. The results showed that vitamin C, IKDCP, and ITP alleviate the spleen tissue lesions of aging model mice induced by D-galactose. The strongest effect was observed for ITP treatment, which resulted in a spleen that was similar in appearance to a normal spleen.

### Expression of mRNA in Mouse Liver, Skin, and Spleen Tissue

**FIGURE 4:**
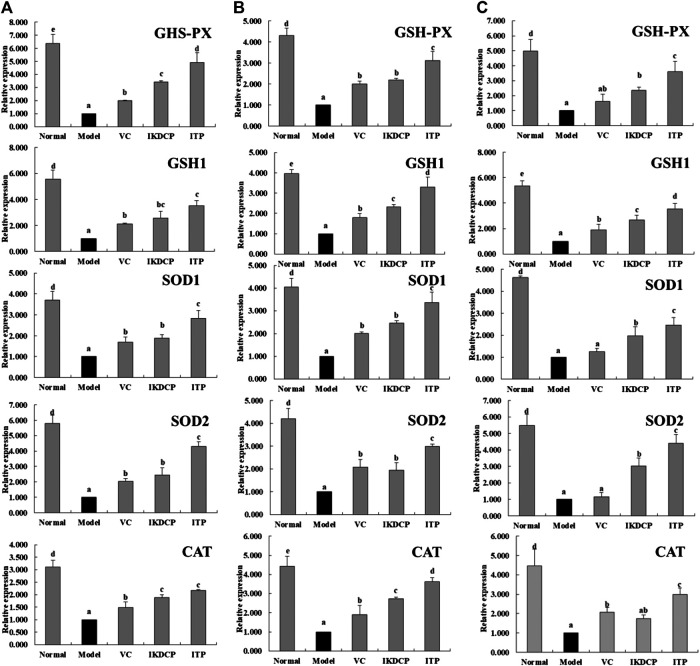
The GSH-PX, GSH1, SOD1, SOD2 and CAT mRNA expression in liver **(A)** skin **(B)** spleen **(C)** tissue of mice. ^a–e^Mean values with different letters in the bar are significantly different (*p* < 0.05) according to Duncan’s multiple-range test. VC: mice treated with vitamin C (100 mg/kg); IKDCP: mice treated with polyphenol of *Ilex kudingcha* (500 mg/kg); ITP: mice treated with polyphenol of insect tea (500 mg/kg).

The mRNA expression of *GHS-PX, GSH1, SOD1, SOD2*, and *CAT* was strongest in the liver, skin, and spleen tissues of normal mice (Figure 4 A, B, C respectively). However, the aging mice (model group) exhibited the opposite expression tendency. After vitamin C, IKDCP, and ITP processing, the expression of *GHS-PX, GSH1, SOD1, SOD2*, and *CAT* in liver, skin, and spleen tissues significantly increased (*p* < 0.05). Vitamin C, IKDCP, and ITP significantly inhibited the down regulation of related anti-oxidant genes. The effect of ITP was stronger, and the expression of *GHS-PX, GSH1, SOD1, SOD2*, and *CAT* in liver, skin, and spleen tissues of aging mice was similar to that of normal mice.

### Expression of Protein in Mouse Liver, Skin, and Spleen Tissue

**FIGURE 5:**
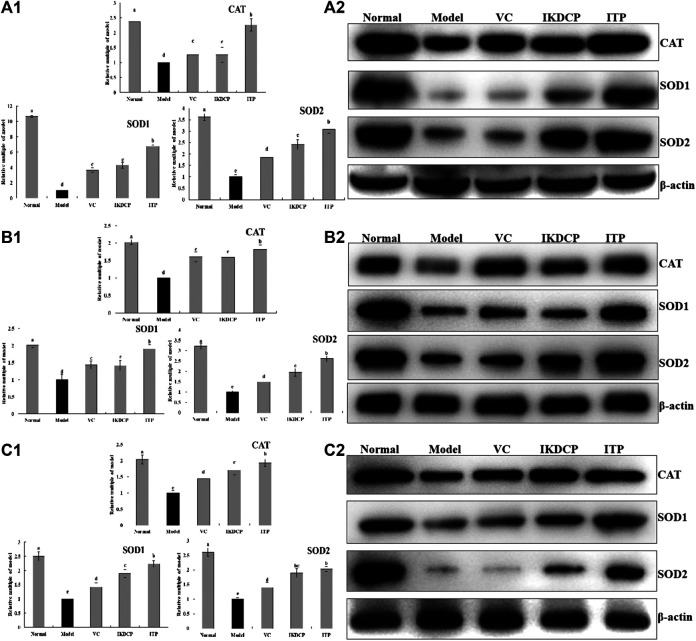
The SOD1, SOD2 and CAT protein expression relative multiple of model **(A)**, protein band **(B)** in liver (1), skin (2), and spleen (3) tissue of mice. ^a–e^Mean values with different letters in the bar are significantly different (*p* < 0.05) according to Duncan’s multiple-range test. VC: mice treated with vitamin C (100 mg/kg); IKDCP: mice treated with polyphenol of *Ilex kudingcha* (500 mg/kg); ITP: mice treated with polyphenol of insect tea (500 mg/kg).

Figures 5 indicate that the protein expression of *CAT, SOD1*, and *SOD2* in liver (1), skin (2), and spleen 3) tissue of the model group was significantly (*p* < 0.05) lower than that of other groups, while the expression of the normal group mice were significantly stronger. Vitamin C, IKDCP, and ITP significantly up regulated *CAT, SOD1*, and *SOD2* expression in the liver, skin, and spleen tissue of aging mice. ITP was more effective than IKDCP or vitamin C. The effect of ITP resulted in the expression of *CAT, SOD1* and *SOD2* in each aging mouse tissue that was similar to that of normal mice.

## Discussion

*Ilex kudingcha* is traditionally used as an herbal medicine in China and Southwest Asia. Studies have shown that *Ilex kudingcha* is rich in polyphenols such as *coffee base quinic acid* (CQA) and its derivatives (Zhu et al., 2009). *Ilex kudingcha* has been shown to have many beneficial functions, including anti-oxidant, anti-obesity, anti-diabetic, anti-inflammatory, cardiovascular, hepatoprotective, and neurological functions (Liu et al., 2009; Thuong et al., 2009; Song et al., 2013). *Insect tea* is a traditional tea drink that is different from ordinary tea, and is consumed for good health and medicinal purposes. *Insect tea* contains 17 mineral elements, including *K, Mg, Ca, Na, Fe, Mn*, and *Zn* are essential trace elements used by the human body. The amounts of *Fe, Zn, Ca*, and *Mg* are higher than those found in some well-known teas, such as *Longjing, Biluochun* and *Tieguanyin* (Xu et al., 2013; Mao et al., 2020). In addition, insect tea is rich in crude protein, crude fiber, fat, tea polyphenols, caffeine, sugar, vitamins, and amino acids. It enhances detoxification, strengthens the stomach and digestion, and exerts free radical scavenging, anti-oxidant, and anti-inflammatory effects (Zhao et al., 2018; Zhu et al., 2019; Zhang et al., 2020). In this study, We made statistics on the previous research reports (Lin C. S. et al., 2018; Svenja et al., 2020; Zhang J. et al., 2020; Zhang W. et al., 2020; Zhu et al., 2019), obtained the active substances that may be contained in *Ilex kudingcha* and Insect tea, and established a database of biologically active ingredients. 33 bioactive components were detected in ITP and IKDCP by UHPLC-QqQ-MS, with the majority being *chlorogenic acid* and its isomers, as well as i*soquercitrin, kaempferol, dihydroquercetin, naringin*.

*Chlorogenic acid* is a *phenolic acid* with an ortho-hydroxyl group on an aromatic residue and is derived from the esterification reaction of *trans-cinnamic acid* (including *caffeic acid, ferulic acid*, and *p-coumaric acid*) with *quinic acid*. There are several subclasses of *chlorogenic acids*, including *neochlorogenic acid, cryptochlorogenic acid, isochlorogenic acid A, isochlorogenic acid B*, and *isochlorogenic acid C*. *Chlorogenic acid* is a secondary metabolite of polyphenols widely present in tea. It has strong antioxidant property, can chelate metal ions and scavenge free radicals, *hypochlorous acid*, *peroxynitrite anions* and *nitric oxide*, and the activity of scavenging free radicals is 10–30 times that of vitamin C (Kono et al., 1997; Lu et al., 2000). In addition, both clinical and basic researches have proved that *chlorogenic acid* is beneficial to health (Naveed et al., 2018; Kumar et al., 2020). As one of the most abundant and functional polyphenols in human diets and drinks, the intake of *chlorogenic acid* is negatively related to the risk of metabolic syndrome and chronic diseases (Lu et al., 2020), it has pharmacological effects such as strong antioxidant effects, lowering blood pressure and blood lipids, protecting liver and gallbladder, antibacterial, anti-inflammatory, and anti-tumor effect (Xia et al., 2019; Li et al., 2019). The results of the current study show that *chlorogenic acid, cryptochlorogenic acid* were present in higher quantities and as the main anti-oxidant components in IKDCP, which matched the finding of a previous report (Wu, et al., 2020). However, the *Caffeic acid*, *isochlorogenic acids A, isochlorogenic acids B*, and *isochlorogenic acids C* were prominent in ITP. It further shows that ITP and IKDCP have strong antioxidant capacity.

There are obvious differences in other biologically active components in ITP and IKDCP. *Isoquercitrin* is a flavonoid compound obtained by the hydrolysis of *rutin*. It is widely present in mulberry leaves, apocynum, white bamboo leaves, and solanum. It is one of the important active ingredients of IKDCP with anti-depressant, anti-inflammatory, anti-oxidant, sedative, and anti-cancer effects (Li et al., 2019). *Kaempferol* is a natural flavonoid with a variety of biological activities, including anti-oxidant and anti-inflammatory effects (Cheng et al., 2018), and its content in ITP is much higher than in IKDCP. *Dihydroquercetin (taxifolin)*, widely present in Pinaceae plants, is a dihydroflavonoid compound. Because it is a polyhydroxy compound, especially 5,7-OH and 3,4-meta-dihydroxy, it confers resistance to oxidation that is stronger than that of general bioflavonoids. As a raw powerful anti-oxidant, it has a variety of biological activities in the human body, including anti-oxidation, scavenging free radicals, anti-virus, anti-inflammatory, vasodilation, and antibacterial effects (Yang et al., 2019). Its content in ITP is obviously higher than that in IKDCP, further indicating that the potential antioxidant activity of ITP must be preferable to that of IKDCP. *Naringin (naringenin-7-O-rutinoside)* is mainly present in citrus peel. Modern therapeutic research has proven that naringin has anti-oxidant, anti-inflammatory, and anti-allergic effects (Funaguchi et al., 2007), it was detected in higher levels in ITP. In summary, although the total content of IKDCP bioactive substances is relatively high, the composition types of IKDCP are reversely small. On the contrary, the total content of ITP is relatively low compared with IKDCP, but it contains a variety of components, and the content of each component is surprisingly high compared that with IKDCP. Therefore, we conclude that the presence of these active substances in ITP may be the main reason why their multiple pharmacological effects.

Continuously injecting D-galactose into animals for a long time will increase the galactose concentration in the animal body, and a large amount of galactitol is then generated by the action of aldose reductase. When galactitol cannot be eliminated in time, it will build up in the body. The normal osmotic pressure of the cell is destroyed, resulting in disorder of the cell's metabolic function and the accumulation of free radicals, which induces the body’s oxidative stress response, and eventually leads to the aging of the organism (Cui et al., 2006; Wei et al., 2005).

The organ index data for mice is one of the important basic indicators of biomedical research. Changes in organ quality can directly reflect the aging of the body. The liver and kidney are metabolic organs in mice, and a decline in their mass directly affects the animal’s metabolic capacity. The spleen is closely linked to the cellular immune response in animals and plays an important role in the immune mechanism. A decline in the quality of the spleen indicates that the organs have atrophied, with a concomitant reduction in their immune function. The organ index can directly reflect the structural changes in the organs, indirectly explaining the function of the organs. Therefore, observing the changes in the mouse organ index has principal reference value for determining whether a mouse is aging (Hsu et al., 2005; Isanga et al., 2008). Studies have demonstrated that when the body ages, many organs and their functions will be degraded, and the heart, liver, spleen, and kidney indexes will decline to some extent (He et al., 2020; Zhang et al., 2020). Parallel research results appeared in this study. The visceral indices of aging model mice were significantly lower than those of the normal group and the vitamin C, IKDCP, and ITP treatment groups, and a more obvious alleviation of mouse organ aging caused by D-galactose was observed after ITP treatment.

In molecular biology, it is believed that with the increase in age, the activity of anti-oxidant enzymes in the body continues to decline, resulting in the inability of oxygen free radicals in the body to be sufficiently removed and the generation of peroxides, which causes the aging and death of cells and accelerates the body's senescence. *SOD, MDA, T-AOC, GSH, NO,* and *GSH-PX* levels are common markers of *in vivo* enzymes and non-enzyme systems that reflect the level of oxidative stress in the body. Studies have shown that when the body ages, the *MDA* content increases and the *SOD* activity decrease. Therefore, the body’s *SOD* activity and *MDA* content can be used as important indicators to measure body aging (Tian et al., 2019).

*SOD* is a particularly important enzyme in the body’s anti-oxidant system. During the aging process of the body, excess superoxide anion radicals are generated. The lifespan of the superoxide anion radicals is relatively long, resulting in induction of the production of hydroxyl radicals and the like, which then cause harm to cells, tissues, and the body. *SOD* activity in the body can reflect the ability of endogenous anti-oxidant enzymes to protect the body. In previous studies, the changes in anti-oxidant enzyme activity and lipid peroxidation during the aging process of the body were measured, and it was confirmed that the decrease in *SOD* activity is closely related to the body's aging (Noblanc et al., 2020). *CAT* is a very important anti-oxidant enzyme in the body that is essential for the regulation of superoxide anion free radicals and hydrogen peroxide levels in the body. *SOD* catalyzes the reaction of superoxide anion free radicals, which results in the generation of hydrogen peroxide, and continuous accumulation of hydrogen peroxide has an inhibitory effect on *SOD*. *CAT* can convert the generated hydrogen peroxide into a non-toxic substance, thereby regulating the balance of hydrogen peroxide and superoxide anion free radicals in the body. *SOD* and *CAT* to coordinate with each other and work together to protect the body (Li et al., 2019).

In the current study, aging induced by D-galactose caused oxidative stress response in the body. The excess production of superoxide anion radicals and hydroxyl radical pairs reduced the body's anti-oxidant enzyme activity. IKDCP and ITP can inhibit this phenomenon, increase anti-oxidant enzyme activity, and restore normal serum and liver tissue levels of *SOD* and *CAT*, thereby delaying the body’s aging. Due to the richness and high content of polyphenols in ITP, plus the minerals, amino acids, and other active substances in the insect tea, a synergistic effect may occur that amplifies the body’s ability to scavenge free radicals, resulting in increased effectiveness for ITP in its action to regulate D-galactose-induced aging as compared to IKDCP.

*NO* is a highly reactive free radical in the body that can relax vascular smooth muscle, inhibit platelet aggregation, and mediate cytotoxic effects and immune regulation. Aberrant production of *NO* is closely related to the occurrence and development of certain diseases. With an increase in the aging of the body, the concentration of *NO* continuously increases (Livak et al., 2001). The lipid peroxidation metabolite *MDA* can damage biological macromolecules such as proteins and nucleic acids, causing the body to age and develop various diseases. The level of *MDA* indirectly reflects the degree of free radical attack on the body cells, and it is another important indicator of the degree of oxidative damage in the body (Si et al., 2019). Reducing the *MDA* content in tissues and serum are of great significance to alleviate oxidative damage. *T-AOC* is a reliable indicator of all the enzymes and non-enzymes in the serum. It can remove active oxygen, maintain the dynamic balance of active oxygen in the internal environment, and balance the state of dynamic redox in the body (Wang C. et al., 2019).

*GSH-PX* is an important anti-oxidant enzyme that is widely present in the body and catalyzes the decomposition of hydrogen peroxide and peroxides. Its active center is selenocysteine (Wang et al., 2016). D-Galactose induces the body to produce excess reactive oxygen species, attacks lipid substances in cells, and generates lipid peroxides, while *GSH-PX* can specifically catalyze the reduction reaction of reduced glutathione to hydrogen peroxide and peroxide, remove harmful peroxide metabolites in the cell, promote the decomposition of hydrogen peroxide, and block the lipid peroxidation chain reaction, thereby protecting the integrity of the cell membrane structure and function. The amount of *GSH-PX* in the serum reflects the body’s oxidative stress and its anti-oxidant capacity (Giardino et al., 2002).

It has been reported that probiotics and tea polyphenols can increase the levels of *MDA* and *NO* in serum, kidney, and liver tissues and decrease the activities of *SOD*, *T-AOC*, and *GSH-PX* caused by D-galactose in aging mice (Li et al., 2020). The same results were achieved in this study. After intragastric administration of IKDCP, ITP, and vitamin C, the levels of *MDA* and *NO* in serum and liver tissue decreased, and the activities of *SOD, T-AOC, GSH*, and *GSH-PX* increased. The parameters of the ITP group basically returned to normal levels, indicating that ITP possessed strong *in vivo* anti-oxidant and health-promoting capabilities. By eliminating free radicals, ITP reduces the loss of anti-oxidant enzymes and increases the body’s anti-oxidant capacity, thus preventing oxidative damage and achieving anti-aging effects.

Oxidative stress is related to the occurrence of various diseases. Free radicals in the body can promote the production and metabolism of arachidonic acid, increase the production of inflammatory intermediates, and promote the production of plasma chemokines that will aggravate inflammation. *IL-1β* and *TNF-α* are produced by macrophages and monocytes. They are the most important cytokines in the early stage of inflammation, and are also known as alert cytokines. They can increase cell permeability and cause inflammatory cells to aggregate, with increasing cell infiltration (Bhatia et al., 2004). *IL-6* is a pleiotropic cytokine, mainly formed by the differentiation of activated T cells and monocyte macrophages, and it has a certain effect in regulating the body’s aging, inflammation, and disease (Maccioni et al., 2009). *IL-10*, which is mainly produced by Th2 cells, inhibits the release of immune-regulating cytokines by Thl cells, and inhibits the release of inflammatory mediators, cytokines, and oxygen free radicals. When induced by D-galactose, mice generate glycosylation terminal irreversible polymers called *AGEs in vivo*. *AGEs* induce the expression of transcription factors *NF-κB*, thereby further upregulating the production of multiple cytokines (Song et al., 1999; Maczurek et al., 2008). With the continuous aging of the mouse body, a large number of *ROS* are activated in the body, which can induce the expression of *TNF-α, IL-1β*, and *IL-6* (Woo et al., 2014). The serum pro-inflammatory factors *TNF-α, IL-1β*, and *IL-6* increased, while the anti-inflammatory factor *IL-10* decreased (Zeng et al., 2020).

The results of the current study show that administering VC, IKDCP, and ITP to aging mice induced by D-galactose can effectively alleviate the oxidative stress pathway, hinder the large increase in inflammatory factors, and lead to normal serum levels of *IL-1β, IL-6, IL-10*, and *TNF-α*. Both IKDCP and ITP reduced the aggregation of inflammatory cells and inhibited the inflammatory response, but a stronger effect was obtained by ITP rather than IKDCP, and ITP is more effectual in regulating D-galactose-induced inflammation.

The process of aging is extremely complex and involves the decreasing function of multiple organs, such as the liver, spleen, and skin tissues. The liver is a major organ that detoxifies potentially harmful chemicals and regulates the environmental stability of the body (Lebel et al., 2011). We observed obvious changes in sinusoidal endothelial cells in aging mice, as well as irregular hepatic cell morphology, untidy arrangement, and eosinophilia and microfocal necrosis that appeared and was accompanied by inflammatory cell infiltration. This is in agreement with the liver tissue of aging mice as reported by Anantharaju (Anantharaju et al., 2019).

The skin is the largest organ in the body, and skin changes are one of the main characteristics that accompany aging. With the aging of the body, the skin undergoes degenerative changes, collagen fibers in the dermal tissue are reduced, the skin is thinner, the water content is reduced, and skin shrinkage occurs (Wang Y. et al., 2019). The spleen is the most important secondary lymphoid organ in the body. It contains nearly one-fourth of all lymphocytes necessary for innate and adaptive immune responses, and it is the center of cellular and humoral immunity (El-naseery et al., 2020). The spleen structure of the oxidatively damaged mice disappeared, the shape was irregular, the red pulp myelinous sinus expanded and was filled with a large number of red blood cells, the white pulp lymphocytes were reduced, the red pulp myelin was narrowed, and the cells were sparsely arranged. Our data show that the liver tissue, skin, and spleen of aging mice induced by D-galactose lose normal structures, but after vitamin C, IKDCP, and ITP treatment, the oxidative damage to the liver, skin, and spleen tissue was alleviated, liver cells were neatly arranged, a small amount of cell necrosis and inflammatory cell infiltration appeared, skin shrinkage appeared to be relieved, and the dermal layer became thick. In the spleen, the boundary between the white pulp region and the red pulp region was rich. The atrophy of the white pulp region is scarce, and the cells at that location are densely arranged. The overall effect from ITP is more optimal as compared to IKDCP and vitamin C.

Studies have shown that aging is related to free radicals generated in organisms, which can react with lipids, proteins, and nucleic acids to produce lipid peroxides, which denature proteins, inactivate enzymes, and break DNA chains. This series of events subsequently causes biofilm damage to the system and allows the disease to develop. Anti-aging related genes and proteins in tissues, such as *Cu/Zn-SOD, Mn-SOD, CAT, GSH1*, and *GSH-PX*, can be used as genetic indicators for detecting oxidative damage to liver, skin, and spleen caused by D-galactose induction (Li et al., 2019; Jin et al., 2020). Previous studies have shown that D-galactose-induced oxidative damage can regulate and restore the free radical enzymes *SOD* and *GSH-PX* in the body (Li et al., 2019). Therefore, by measuring the expression levels of these important anti-oxidant-related genes and proteins, the anti-oxidant effects of IKDCP and ITP were determined, and their anti-oxidant capacity was compared. Compared with the model group, IKDCP and ITP upregulated the expression of *Cu/Zn-SOD, Mn-SOD, CAT, GSH1*, and increased *GSH-PX* mRNA and protein, indicating that the free radicals produced by oxidative metabolism in the liver, skin, and spleen tissue of aging mice induced by D-galactose can be eliminated. An anti-oxidant effect was observed after ITP treatment, which upregulated the expression of anti-oxidant-related genes and proteins so that they were similar to that of normal organisms, which is consistent with the results of Guo et al. (2020) and Yi et al. (2019).

In conclusion, we evaluated there *in vivo* protective effects on D-galactose-induced aging mice, and their potential bioactive substances. The results showed that the anti-aging effects of IKDCP and ITP (500 mg/kg) include the regulation of viscera indices of major organs; improvement in liver, skin, and spleen tissue morphology; a decrease in inflammatory cytokine production; upregulation of *SOD, CAT, GSH, GSH-PX*, and *T-AOC*, and downregulation of *NO* and *MDA* levels in serum and liver tissue; reductions in the concentration of pro-inflammatory factors (*IL-6, TNF-α*, and *IL-1β*); and increases in the concentration of anti-inflammatory factor *IL-10*. RT-qPCR and western blot assay also showed that IKDCP and ITP affect anti-aging by regulating the gene and protein expression of *GSH-PX, GSH1, SOD1, SOD2*, and *CAT*. In addition, these biological activities of IKDCP and ITP may be conferred by their chemical constituents, including flavonoids and phenolic acids. The 33 compounds detected by UHPLC-QqQ-MS have previously been shown to have a variety of biological activities, including anti-aging, anti-inflammatory, and anti-cancer. However, due to the substantial differences in the content of the primary chemical components in IKDCP and ITP, the effects are also different. The overall results indicate that ITP is more effective for treating aging mice with oxidative damage that was induced by D-galactose.

## Author’s Note

*Ilex kudingcha* and insect tea are stored for posterity and publicly accessible in Chongqing Collaborative Innovation Center for Functional Food, Chongqing University of Education, China, and the *Ilex kudingcha* and insect tea can be obtained from the corresponding authors.

## Data Availability Statement

The original contributions presented in the study are included in the article/Supplementary Material, further inquiries can be directed to the corresponding author.

## Ethics Statement

The protocol for these experiments was approved by the Ethics Committee of Chongqing Collaborative Innovation Center for Functional Food (201905030B), Chongqing, China.

## Author Contributions

JM performed the majority of the experiments and wrote the manuscript; FY finished revising and proofreading this paper; FT, XZ, YP, and XL contributed to the data analysis; XZ designed and supervised the study, and checked the final manuscript.

## Funding

This research was funded by Chongqing University Innovation Research Group Project (CXQTP20033) and the Introduction of High-level Person-nel Research Start-up Fund of Chongqing University of Education (2013BSRC001), China.

## Conflict of Interest

The authors declare that the research was conducted in the absence of any commercial or financial relationships that could be construed as a potential conflict of interest.

## Abbreviations

IKDCP, *Ilex kudingcha* polyphenol; ITP, insect tea polyphenol; UHPLC-QqQ-MS, ultra high-performance liquid chromatography-triple quadrupole mass spectrometry; SOD, superoxide dismutase; CAT, catalase; GSH, glutathione; GSH-PX, glutathione peroxidase; T-AOC, total antioxidant capacity; NO, nitric oxide; MDA, mobile device assistant; SOD1, Cu/Zn superoxide dismutase; SOD2, Mn superoxide dismutase; GAPDH, glyceraldehyde-3-phosphate dehydrogenase; TNF-α, tumor necrosis factor-α; IL-1β, andinterleukin-1β; IL-6, interleukin-6; IL-10, interleukin-10; RT-qPCR, real-time quantitative polymerase chain reaction; ESI, electrospray ionization; MRM, multiple reaction monitoring; DPPH, 2, 2-diphenyl-1-picrylhydrazyl; VC, vitamin C; ABTS, 2, 2′-azino-bis (3-ethylbenzothiazoline-6-sulphonic acid); RIPA, radioimmunoprecipitation; PMSF, phenylmethylsulfonyl fluoride; TEMED, N,N,N,N-tetramethylethylenediamine; APS, ammonium persulfate; PVDF, polyvinylidene fluoride; TBST, Tris-buffered saline containing 0.1% Tween-20; AGEs, advanced glycation end-products; ROS, reactive oxygen species; NF-κB, nuclear factor *κ*-light-chain-enhancer of activated B cells.
